# Navigating the Challenges of Conducting Training Programs for Serious Mental Disorders for different stakeholder in Slum in Bangladesh

**DOI:** 10.1192/j.eurpsy.2025.919

**Published:** 2025-08-26

**Authors:** D. T. Rashid Soron, K. S. Azmery, F. Ahmed, M. Rahman, M. Belaluzzaman, A. A. Mamun, T. Consortium

**Affiliations:** 1 Telepsychiatry Research and Innovation Network, Dhaka, Bangladesh; 2 University of Warwick, Coventry, United Kingdom

## Abstract

**Introduction:**

Mental health treatment gap is over 92% in Bangladesh. The situation is more disappointing for those living in the slums in this country where basic health care and other facilities are limited. Due to the lack of easy and affordable access to biomedical care, the slum populations predominantly seek mental health care from traditional and faith-based healers, community health workers (CHWs), and local medicine sellers for serious mental disorders. In the TRANSFORM research, we are working with these 4 important stakeholders and codeveloped two different training programs clustering them in two different groups. In one group, we provided training for traditional and faith based healers and in another group community health workers and medicine sellers and a total 153 people received our 3 days long codeveloped training in 4 batches.

**Objectives:**

We aimed to document the different types of challenges; we encounter to conduct 3 days long training program on serious mental disorders in two groups in the 4 batches.

**Methods:**

A mixed-methods approach was employed to document challenges faced in three stages: pre-training assessment, during-training documentation, and post-training evaluation. During the pre and post feedback assessment we asked participants to provide feedback on specific challenges they faced. The process documentation and event notes throughout the program were collected. After training of each batch, we organized FGDs to gather qualitative insights about the challenges and separate FGD was organized with the facilitators, organizing team members and researchers to get their views also. Thematic analysis was performed on qualitative data, and descriptive statistics were used to assess quantitative findings.

**Results:**

The challenges were clustered in 5 key thematic areas 1) challenge related to manage the diversity of the training participants within the group in terms of age, educational level, religions, 2) challenge related to time and schedule management – due to the rain, traffic jam, ramadan and eid holidays, 3) sociocultural and stigma related challenges: such as old women were unwilling to take part in role 4) Challenge emerged due to disaster **:** A devastating fire ravaged the slum leaving thousands homeless during the training, in addition the heavy rain and storm, 5) political and policy related challenges - During the fourth training batch, political unrest led to roadblocks and safety concerns, making it difficult for participants and facilitators to attend the training.

**Conclusions:**

The understanding and early identification of challenges might be helpful in successful implementation of psychiatry training in different settings.

**Disclosure of Interest:**

None Declared

